# Diagnostic utility of a conventional MRI-based analysis and texture analysis for discriminating between ovarian thecoma-fibroma groups and ovarian granulosa cell tumors

**DOI:** 10.1186/s13048-022-00989-z

**Published:** 2022-05-25

**Authors:** Keita Nagawa, Tomoki Kishigami, Fumitaka Yokoyama, Sho Murakami, Toshiharu Yasugi, Yasunobu Takaki, Kaiji Inoue, Saki Tsuchihashi, Satoshi Seki, Yoshitaka Okada, Yasutaka Baba, Kosei Hasegawa, Masanori Yasuda, Eito Kozawa

**Affiliations:** 1 Department of Radiology, Saitama Medical University, 38 Morohongou, Moroyama-machi, Iruma-gun, Saitama, Japan; 2 Department of Radiology, Tokyo Metropolitan Cancer and Infectious Diseases Center Komagome Hospital, 3-18-22 Honkomagome, Bunkyo-ku, Tokyo, Japan; 3 Department of Gynecology, Tokyo Metropolitan Cancer and Infectious Diseases Center Komagome Hospital, 3-18-22 Honkomagome, Bunkyo-ku, Tokyo, Japan; 4 Department of Diagnostic Imaging, Saitama Medical University International Medical Center, 1397-1 Yamane, Hidaka city, Saitama, Japan; 5 Department of Gynecologic Oncology, Saitama Medical University International Medical Center, 1397-1 Yamane, Hidaka city, Saitama, Japan; 6 Department of Diagnostic Pathology, Saitama Medical University International Medical Center, 1397-1 Yamane, Hidaka city, Saitama, Japan

## Abstract

**Objective:**

To evaluate the diagnostic utility of conventional magnetic resonance imaging (MRI)-based characteristics and a texture analysis (TA) for discriminating between ovarian thecoma-fibroma groups (OTFGs) and ovarian granulosa cell tumors (OGCTs).

**Methods:**

This retrospective multicenter study enrolled 52 patients with 32 OGCTs and 21 OTFGs, which were dissected and pathologically diagnosed between January 2008 and December 2019.

MRI-based features (MBFs) and texture features (TFs) were evaluated and compared between OTFGs and OGCTs. A least absolute shrinkage and selection operator (LASSO) regression analysis was performed to select features and construct the discriminating model. ROC analyses were conducted on MBFs, TFs, and their combination to discriminate between the two diseases.

**Results:**

We selected 3 features with the highest absolute value of the LASSO regression coefficient for each model: the apparent diffusion coefficient (ADC), peripheral cystic area, and contrast enhancement in the venous phase (VCE) for the MRI-based model; the 10th percentile, difference variance, and maximal correlation coefficient for the TA-based model; and ADC, VCE, and the difference variance for the combination model. The areas under the curves of the constructed models were 0.938, 0.817, and 0.941, respectively. The diagnostic performance of the MRI-based and combination models was similar (*p* = 0.38), but significantly better than that of the TA-based model (*p* < 0.05).

**Conclusions:**

The conventional MRI-based analysis has potential as a method to differentiate OTFGs from OGCTs. TA did not appear to be of any additional benefit. Further studies are needed on the use of these methods for a preoperative differential diagnosis of these two diseases.

**Supplementary Information:**

The online version contains supplementary material available at 10.1186/s13048-022-00989-z.

## Background

Ovarian granulosa cell tumors (OGCTs) and ovarian thecoma-fibroma groups (OTFGs) are both uncommon neoplasms that originate from the sex cord-stromal cells of the ovary [1, 2]. The clinical manifestations of these two groups overlap, and include vaginal bleeding, endometrial hyperplasia, and carcinoma, which are related to excessive estrogen production [3, 4]. OGCTs are regarded as a low-grade malignancy, but with a modest risk of metastasis or the potential to recur years after the initial diagnosis [5, 6]. In the advanced stages of OGCT, chemotherapy is recommended as adjuvant treatment [6]. Follow-ups are also recommended for all patients with OGCTs due to the modest to high rate of recurrence (17–50%). In contrast, OTFGs are benign tumors that rarely metastasize or recur and often may be treated conservatively [7]. Therefore, an accurate preoperative diagnosis between OGCTs and OTFGs has significant implications for clinical treatment and management strategies.

Magnetic resonance imaging (MRI) is a feasible method for providing important diagnostic information on pelvic lesions, such as OGCTs and OTFGs. A high signal intensity on T1-weighted images (T1WI) representing hemorrhage, a high signal intensity on diffusion-weighted imaging (DWI), a low apparent diffusion coefficient (ADC) value, and moderate enhancement are characteristic MRI features of OGCTs [8–10]. In contrast, a low signal intensity on T1WI and T2-weighted images (T2WI) indicating a fibrous tissue component, a high ADC value, and minimal or mild enhancement are the predominant features of OTFGs [11, 12]. Although previous studies reported characteristic MRI findings for these two tumors, quantitative or semi-quantitative assessments with MRI have been limited.

A texture analysis (TA) is an image analysis technique that allows for the quantification of image characteristics using the distribution of pixels and their surface intensity or patterns [13, 14]. These image characteristics are based on the microstructures of a background tissue and are sometimes imperceptible to the human visual system [13]. TA has been applied to a number of medical image assessments, including oncologic imaging [15, 16], neuroimaging [17, 18], and musculoskeletal imaging [19, 20]. In pelvic MRI on ovarian sex cord-stromal tumors, recent radiomics studies reported the differentiation of OTFGs from uterine fibroids in the adnexal area [21]. A discriminative analysis of OGCTs and OTFGs using imaging-based models and TA-based models was conducted by Li et al. [22]. Although there are clinical merits to predictive models for these adnexal lesions, the number of related studies is still limited, which may be due to the rarity of these diseases.

Therefore, the present study was performed to assess MRI-based features (MBFs) and texture features (TFs), identify the most favorable features for discriminating between OGCTs and OTFGs, and evaluate the combined diagnostic performance of MBFs and TFs.

## Methods

This was a retrospective multicenter study; each Institutional Review Board provided approval, and informed consent was obtained in the form of an opt-out on the website. All experiments were performed in accordance with the relevant guidelines and regulations.

Data were collected from the Saitama Medical University International Medical Center (SMUIMC) and Tokyo Metropolitan Komagome Hospital (TMKH). We searched their pathology databases for consecutive patients with pathologically confirmed OTFGs and OGCTs between January 2008 and December 2019.

**Fig. 1:**
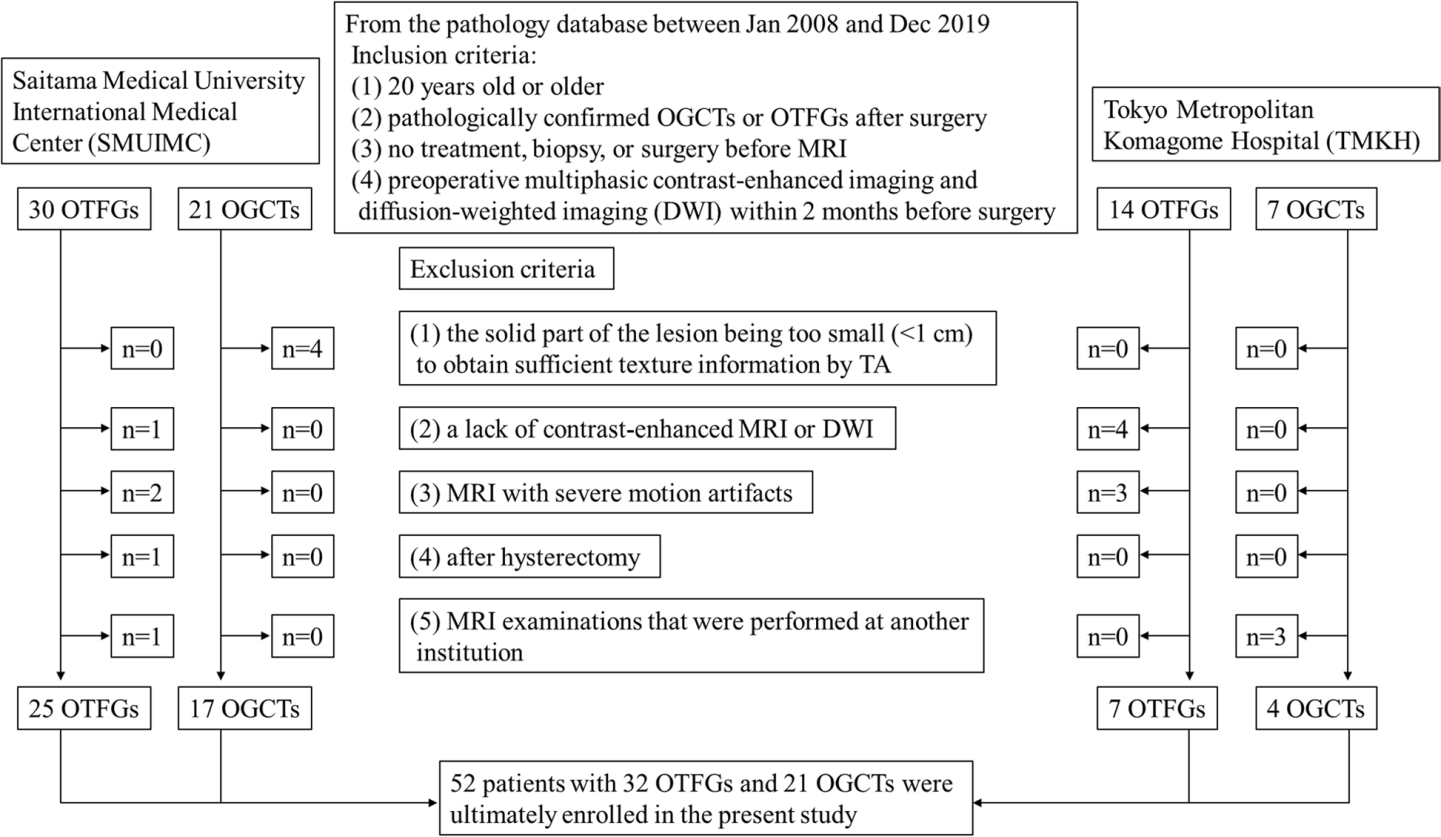
Flowchart of inclusion and exclusion criteria

Inclusion and exclusion criteria were summarized as a flow chart shown in Fig. 1. Based on these criteria, 52 patients with 32 OTFGs and 21 OGCTs were ultimately enrolled in the present study.

### MRI examination

MRI was performed using the 3.0-T system (Achieva, Philips (SMUIMC); and Skyra, Siemens Healthcare (TMKH)). The principal MRI protocol included T2WI in the axial plane, T1WI in the axial plane, DWI in the axial plane, and multiphasic contrast-enhanced fat-suppressed T1WI in the axial, sagittal, or coronal plane. Representative scanning sequences and parameters are shown in Table 1. An intravenous injection of 20 mg of butyl-scopolamine was administered to all patients to relax the bowel wall and reduce peristaltic bowel movement before MRI examinations.

**Table 1:** Representative scanning sequences and parameters

3.0-T Achieva, Philips (Saitama Medical University International Medical Center)						
Sequence	TR/TE (msec)	Matrix	NEX	FOV (cm^2^)	Slice thickness (mm)	Flip Angle (degree)
T2WI	3546–5598/100	352 × 272	2	28 × 32	5–6	90
T1WI	180–448/1.15–2.3	224 × 180	2	28 × 32	5–6	75
DWI	6250–8750/75	112 × 168	2	32 × 36	5–6	90
(*b* = 0, 500, 1,000 s/mm^2^)						
CE-MRI	3.1–3.4/1.54–1.65	256 × 181	1	28 × 32	3–4	12
(multiphasic 3D gradient echo sequence (eTHRIVE); 35, 65, 95, and 125 s after the intravenous injection of 0.1 mmol/kg meglumine gadoterate)						
3.0-T Skyra, Siemens Healthcare (Tokyo Metropolitan Komagome Hospital)						
Sequence	TR/TE (msec)	Matrix	NEX	FOV (cm^2^)	Slice thickness (mm)	Flip Angle (degree)
T2WI	3800–4200/87–91	384 × 269	1	38 × 38	5–6	120
T1WI	520–617/11–13	384 × 269	1	38 × 38	5–6	120
DWI	4000–5700/69–70	128 × 59	1	16 × 26	5–6	90
(*b* = 0, 1,000 s/mm^2^)						
CE-MRI	3.62–3.72/1.3	256 × 151	1	17 × 26	3–4	10
(multiphasic 3D gradient echo sequence (t1_vibe_fs_ce); 30, 60, 90, and 120 s after the intravenous injection of 0.1 mmol/kg meglumine gadoterate)						

*CE* contrast enhancement, *DWI* diffusion-weighted imaging, *FOV* field of view, *NEX* number of excitations, *T1WI* T1-weighted imaging, *T2WI* T2-weighted imaging, *TE* echo time, *TR* repetition time. An intravenous injection of 20 mg of butyl-scopolamine was administered to all patients to relax the bowel wall and reduce peristaltic bowel movement before MRI examinations

### Conventional MRI-based analysis

The evaluation of MBFs was performed by two radiologists (K.I. and K.N.) with 20 and 6 years of experience in gynecological imaging who were blinded to the pathological results. The two radiologists independently assessed MBFs, which were used for inter-reader reproducibility tests. They then reached a consensus for any discrepancies to be resolved and consensus data were used in the construction of classification models.

The following MBFs of the two types of diseases were recorded:

- Lesion size: the maximum lesion diameter of the three dimensions measured.
- Morphology:
  - Multiple nodular: may be divided into many nodules or locules
  - Oval: may be approximated as a round or ellipse shape
  - Shallow lobulated: may consist of lobules, not having a nodular appearance
- Capsule: the presence of a uniformly thin layer covering the whole lesion.
- Solid and cystic components:
  - Solid: without cystic degeneration found by the naked eye
  - Predominantly solid: the area of cystic degeneration was < 1/3 of the total area
  - Solid and cystic: the area of cystic degeneration was 1/3–2/3 of the total area
  - Predominantly cystic: the area of cystic degeneration was > 2/3 of the total area
- Cystic pattern:
  - None
  - Mainly large cysts: the area of large cysts was > 1/2, large cysts were ≥ 1 cm
  - Mainly small cysts: the area of small cysts was > 1/2, small cysts were < 1 cm
  - Mixed: the areas of large and small cysts were almost equal
- Cyst size: the longest diameter of the cystic region.
- Peripheral cystic area: the presence of cystic areas in the peripheral zone of the lesion.
- Hemorrhagic component: the presence of a high signal intensity on T1WI within the lesion.
- Endometrial hyperplasia: an endometrial thickness of more than 16 and 5 mm in premenopausal and postmenopausal women, respectively.
- Uterine fibroids or adenomyosis: the presence of uterine nodules or ill-defined thickening of the myometrium with small foci of hemorrhage or cystic changes.
- Pelvic fluid:
  - None
  - Small: ascites localized in the pelvic cavity
  - Large: ascites extending beyond the pelvic cavity into the peritoneal cavity
- T1WI, T2WI, and DWI: the signal intensity in solid regions was examined.
  - Hypo-, iso-, or hyperintense relative to the uterine myometrium
- ADC value: the mean value within solid regions was obtained after three measurements.
- Contrast enhancement: the degree of contrast enhancement within the lesion was examined in the arterial, venous, and delayed phases.
  - Minimal: clearly less than the myometrium
  - Mild: less than the myometrium
  - Moderate: similar to the myometrium
  - Marked: more than the myometrium

### TA

**Fig. 2:**
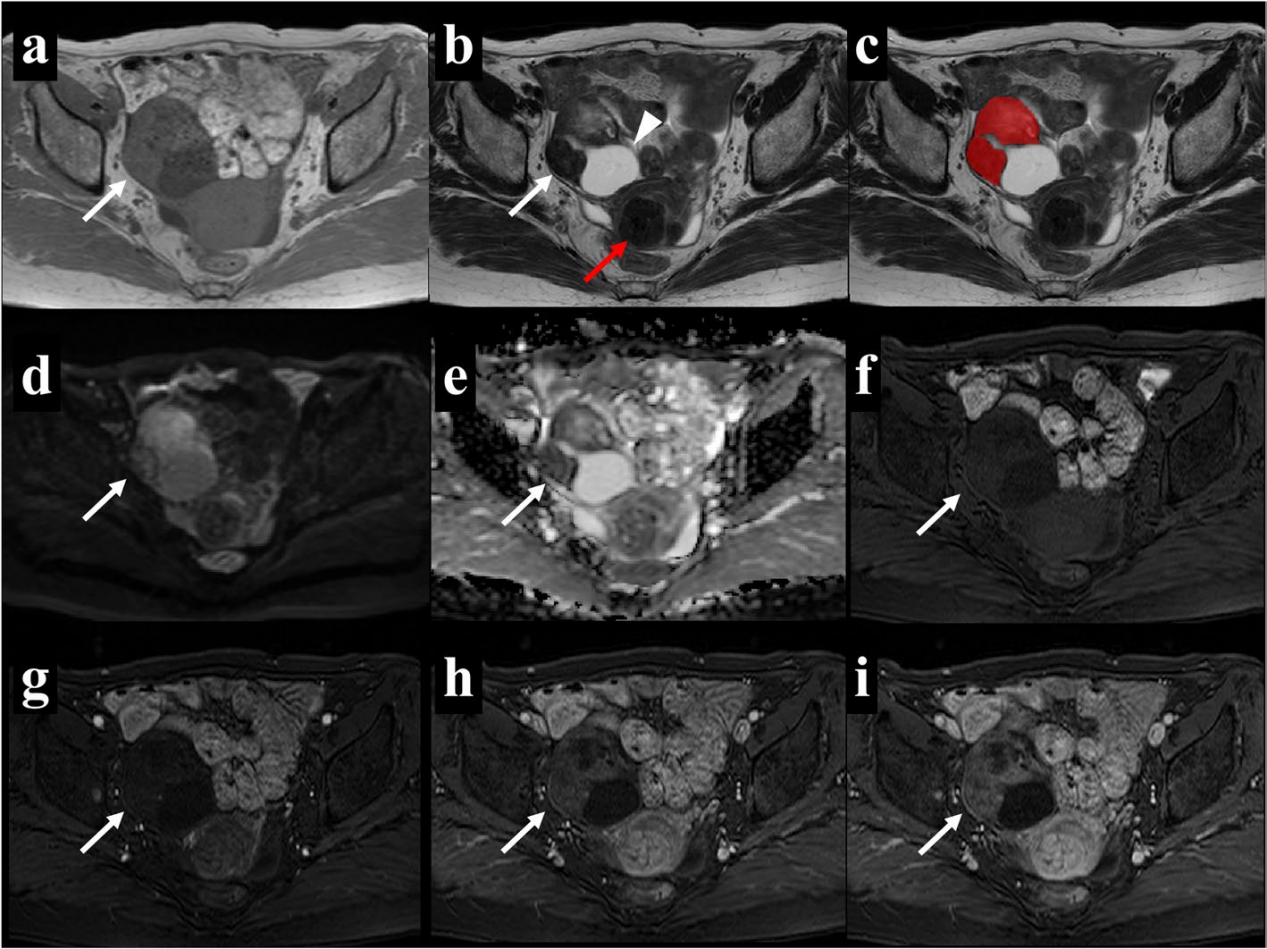
A 66-year-old woman with right ovarian fibrothecoma. Axial T1WI (**a**) showed an isointense solid mass and axial T2WI (**b**) showed a heterogeneous solid mass with a cystic change in the dorsal part (arrowhead). Uterine fibroids were present (red arrow). The region of interest (ROI) in the texture analysis (**c**) was delineated to contain the outline borders of the solid region of the tumor, and the cystic region was avoided as much as possible. Axial DWI (**d**) showed a hyperintense solid area, and the apparent diffusion coefficient (ADC) map (**e**) showed that the average ADC value of this area was 1.31 × 10^−3^ mm^2^/s. In multiphasic contrast-enhanced fat-suppressed T1-weighted MRI (**f**-**i**), the mass showed minimal enhancement in the arterial and venous periods (**g** and **h**, respectively) as well as heterogeneous mild enhancement in the delayed period (**i**)

**Fig. 3:**
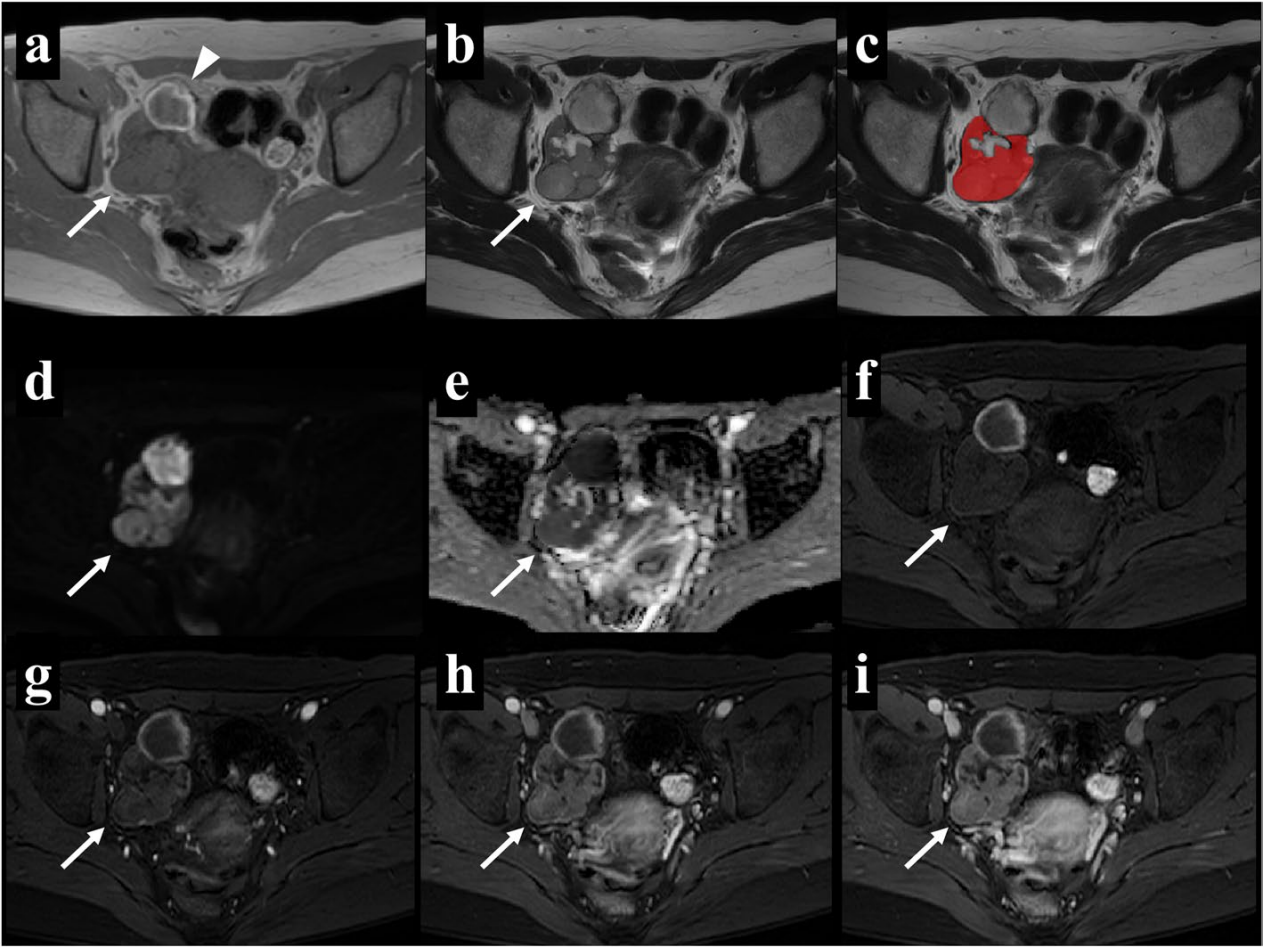
A 45-year-old woman with a right ovarian adult granulosa cell tumor. Axial T1WI (**a**) showed a hemorrhagic change in the dorsal part (arrowhead) and axial T2WI (**b**) showed a homogeneous isointense solid region with a partial cystic change. The region of interest (ROI) in the texture analysis (**c**) was delineated to contain the outline borders of the solid region of the tumor, and the cystic region was avoided as much as possible. Axial DWI (**d**) showed a hyperintense solid area, and the apparent diffusion coefficient (ADC) map (**e**) showed that the average ADC value of this area was 0.84 × 10^−3^ mm^2^/s. In multiphasic contrast-enhanced fat-suppressed T1-weighted MRI (**f**-**i**), the mass mainly showed mild enhancement in all contrast phases

To avoid data heterogeneity bias, all MRI data were subjected to imaging normalization (the intensity of the image was scaled to 0–100), resampled to the same resolution (3 × 3 × 3 mm), and discretized before feature extraction. Segmentation was performed using open-source software (ITK-SNAP version 3.8.0). One slice of axial T2WI that harbored the largest solid region of the tumor was selected for each case. A radiologist with 6 years of experience (K.N.) selected an appropriate slice and then asked two radiologists, both with 3 years of experience (T.K. and F.Y.), to draw on the identified slice. An irregular two-dimensional ROI was manually drawn to contain the outline borders of the solid region of the tumor on each selected image, and the cystic region was avoided as much as possible (see Fig. 2c or 3c). All three radiologists were blinded to clinical information. The ROI data delineated by T.K. and F.Y. were used for an inter-reader reproducibility test, while those delineated by T.K. were used to construct classification models.

TFs were extracted using the open-source python package (PyRadiomics version 2.1.0 (www.radiomics.io/pyradiomics.html)). Extracted TFs were as follows: 18 first-order statistics, 24 Gy-level co-occurrence matrix (GLCM), 16 Gy-level run-length matrix, 16 Gy-level size zone matrix, 14 Gy-level dependence matrix, and 5 neighboring gray-tone difference matrix features.

### Statistical analysis

Quantitative data were summarized in our dataset as means ± standard deviations. Qualitative data were summarized as the total number of cases in our dataset (or percentages). In univariate analyses, the Mann–Whitney U test was used for quantitative data, while Fisher’s exact test or the *x*^2^ test was used for qualitative data.

Before the consecutive statistical analysis, all numeric values were standardized using the Python scikit-learn package “RobustScaler” to minimize the effects associated with differences in the MRI machines and protocols between two centers.

To construct discriminant models that differentiate between OTFGs and OGCTs, we performed feature selection. We adopted the least absolute shrinkage and selection operator (LASSO) algorithm, which may be a suitable method for the regression of high dimensional data with a small sample size, such as that in the present study [23]. All variables were included in each attempt of feature selection, and their LASSO regression coefficients were obtained by tenfold cross-validation. To avoid over-fitting, only three features with the highest absolute values of the LASSO regression coefficient were selected each time. Model development was conducted using a linear discriminant analysis. ROC analyses were performed on independent discriminative features to assess the overall diagnostic performance of the discriminant models.

We constructed the MRI-based model (using selected MBFs), TA-based model (using selected TFs), and combination model (using the best features selected from all MBFs and TFs), and compared their performance. In consideration of the small sample size, we adopted tenfold cross-validation repeated 100 times to construct the final model. The performance of classifiers was evaluated by the area under the curve (AUC). Accuracy, sensitivity, specificity, precision, and the F-measure were calculated based on the confusion matrix of classification results.

Interobserver reproducibility was evaluated for each feature; Kappa values were used in the assessment of qualitative data among MBFs (k ≥ 0.75 was indicative of almost perfect agreement): the intraclass correlation coefficient (ICC) was measured in the assessment of quantitative data among MBFs and TFs (ICC ≥ 0.75 and lower 95%CI ≥ 0.6 indicated good reproducibility). The collinearity status between each feature was analyzed using Pearson’s correlation coefficient (r ≥ 0.7 indicated high collinearity).

Statistical analyses were performed using an opensource software package (Python scikit-learn 0.22.1). Statistical values of *p* < 0.05 were considered to be significant.

## Results

### Clinical characteristics

The clinical characteristics of all of the lesions examined are summarized in Table 2. No significant differences were observed in variables, such as age, side of the lesion, body mass index, the menopausal status, parity, or clinical symptoms, between OTFGs and OGCTs.

**Table 2:** Demographic and clinical characteristics of study patients

	OTFG			OGCT			
Center	SMUIMC (*n* = 25)	TMKH (*n* = 7)	Total (*n* = 32)	SMUIMC (*n* = 17)	TMKH (*n* = 4)	Total (*n* = 21)	*p*-value
Age (years)^a^, mean ± SD	58.2 ± 14.6	63.3 ± 10.9	59.3 ± 14.1	56.9 ± 14.2	60.0 ± 9.7	57.5 ± 13.5	0.478
Side of the lesion^c^							0.257
Right	12*	3	15 (46.9%)	10	4	14 (66.7%)	
Left	13*	4	17 (53.1%)	7	0	7 (33.3%)	
BMI^a^, mean ± SD	22.3 ± 3.4	24.1 ± 3.0	22.7 ± 3.4	22.2 ± 1.9	22.1 ± 1.5	22.2 ± 1.8	0.841
Menopausal status^b^							1.000
Premenopause	6	0	6 (18.7%)	4	0	4 (19.0%)	
Postmenopause	19	7	26 (81.3%)	13	4	17 (81.0%)	
Parity^b^							0.384
Multipara	21	6	27 (84.4%)	17	3	20 (95.2%)	
Nullipara	4	1	5 (15.6%)	0	1	1 (4.8%)	
Clinical symptoms^a^							0.380
Asymptomatic	9	2	11 (34.4%)	3	1	4 (19.0%)	
Pelvic pain	3	1	4 (12.5%)	1	0	1 (4.8%)	
Urinary frequency	0	0	0 (0.0%)	0	1	1 (4.8%)	
Vaginal bleeding	1	1	2 (6.3%)	7	1	8 (38.1%)	
Abdominal distension /discomfort	12	3	15 (46.9%)	6	1	7 (33.3%)	
Pathology							N/A
Fibroma	6	6	12 (37.5%)				
Fibrothecoma	14	0	14 (43.8%)				
Thecoma	3	0	3 (9.4%)				
Cellular fibroma	2	1	3 (9.4%)				
Adult-type GCT							
FIGO stage Ia				10	4	14 (66.7%)	
Ic				3	0	3 (14.3%)	
IIa				1	0	1 (4.8%)	
IIc				1	0	1 (4.8%)	
IIIc				2	0	2 (9.5%)	

Except where otherwise indicated, data are the number (%) of patients. *BMI* body mass index, *OTFG* ovarian thecoma-fibroma group, *OGCT* ovarian granulosa cell tumor, *SD* standard deviation, *SMUIMC* the Saitama Medical University International Medical Center, *TMKH* Tokyo Metropolitan Komagome Hospital. N/A not assessed

^a^Data were tested using the Mann–Whitney U test

^b^Data were tested using Fisher’s exact test

^c^Data were tested using the chi-squared test

^*^Lesions on both sides of the ovaries were included

### Conventional MRI-based characteristics

MRI-based characteristics were compared between OTFGs and OGCTs, as shown in Tables 3 and 4. Representative cases of the two diseases are shown in Figs. 2 and 3. The univariate analysis identified the following ten variables: capsule, solid and cystic components, peripheral cystic areas, the cystic pattern, intratumoral hemorrhage, pelvic fluid, the ADC value, and contrast enhancement in the arterial, venous, and delayed phases, as significant factors for differentiating between the two disease groups (*p* < 0.05).

**Table 3:** MRI-based characteristics of study patients

		OTFG (*n* = 32)	OGCT (*n* = 21)	Univariate analysis (*p*)	LASSO estimate	*Κ*-value
Lesion size (cm), mean ± SD		12.1 ± 5.3	11.8 ± 7.5	0.331 ^a^	-0.071	0.927 ^d^
Morphology				0.472 ^a^	-0.034	0.758
Multiple nodular		10 (31.3%)	8 (38.1%)			
Oval		13 (40.6%)	9 (42.9%)			
Shallow lobulated		9 (28.1%)	4 (19.0%)			
Capsule				0.004 ^b^	0.033	0.903
Absent		13 (40.6%)	1 (4.8%)			
Present		19 (59.4%)	20 (95.2%)			
Solid and cystic components				0.013 ^a^	0.000	0.942
Solid		13 (40.6%)	1 (4.8%)			
Partly cystic		7 (21.9%)	8 (38.1%)			
Solid and cystic		8 (25.0%)	5 (23.8%)			
Partly solid		4 (12.5%)	7 (33.3%)			
Peripheral cystic areas				< 0.001 ^b^	0.097	0.762
Absent		16 (50.0%)	1 (4.8%)			
Present		16 (50.0%)	20 (95.2%)			
Cyst size (cm), mean ± SD		4.2 ± 5.9	5.0 ± 4.5	0.135 ^a^	0.000	0.985 ^d^
Cystic pattern				0.014 ^a^	0.000	0.903
None		13 (40.6%)	1 (4.8%)			
Mainly large		12 (37.5%)	11 (52.4%)			
Mainly small		3 (9.4%)	6 (28.6%)			
Mixed		4 (12.5%)	3 (14.3%)			
Hemorrhagic component				0.004 ^c^	0.000	0.958
Absent		26 (81.2%)	8 (38.1%)			
Present		6 (18.8%)	13 (61.9%)			
Endometrial hyperplasia				0.671 ^b^	0.002	0.899
Absent		29 (90.6%)	3 (9.4%)			
Present		18 (85.7%)	3 (14.3%)			
Uterine fibroid or adenomyosis				0.739 ^b^	0.000	0.801
Absent	11 (34.4%)		21 (42.9%)			
Present	9 (65.6%)		12 (57.1%)			
Pelvic fluid				0.013 ^a^	0.000	0.828
None	2 (6.3%)		0 (0.0%)			
Small	8 (25.0%)		15 (71.4%)			
Large	22 (68.8%)		6 (28.6%)			

Except where otherwise indicated, data are the number (%) of patients. *LASSO* least absolute shrinkage and selection operator, *OTFG* ovarian thecoma-fibroma group, *OGCT* ovarian granulosa cell tumor, *SD* standard deviation

^a^Data were tested using the Mann–Whitney U test

^b^Data were tested using Fisher’s exact test

^c^Data were tested using the chi-squared test

^d^The intra-class correlation (ICC) was calculated instead because of continuous variables

**Table 4:** MRI-based characteristics of study patients

	OTFG (*n* = 32)	OGCT (*n* = 21)	Univariate analysis (*p*)	LASSO estimate	*Κ*-value
ADC value, (× 10^−3^ mm^2^/s) mean ± SD	1.16 ± 0.36	0.77 ± 0.28	< 0.001 ^a^	-0.107	0.842 ^b^
T1WI			0.059 ^a^	0.016	0.785
Hypointensity	5 (15.6%)	3 (14.3%)			
Isointensity	26 (81.3%)	12 (57.1%)			
Hyperintensity	0 (0.0%)	3 (14.3%)			
Mixed	1 (3.1%)	3 (14.3%)			
T2WI			0.205 ^a^	0.000	0.877
Hypointensity	10 (31.3%)	1 (4.8%)			
Isointensity	3 (9.4%)	1 (4.8%)			
Hyperintensity	7 (21.9%)	15 (71.4%)			
Mixed	12 (37.5%)	4 (19.0%)			
DWI			0.277 ^a^	0.081	0.814
Hypointensity	5 (15.6%)	0 (0.0%)			
Isointensity	3 (9.4%)	0 (0.0%)			
Hyperintensity	12 (37.5%)	16 (76.2%)			
Mixed	12 (37.5%)	5 (23.8%)			
Contrast enhancement					
Arterial phase			< 0.001 ^a^	0.000	0.911
Minimal	26 (81.3%)	4 (19.0%)			
Mild	5 (15.6%)	7 (33.3%)			
Moderate	1 (3.1%)	9 (42.9%)			
Severe	0 (0.0%)	1 (4.8%)			
Venous phase			< 0.001 ^a^	0.267	0.930
Minimal	19 (59.4%)	0 (0.0%)			
Mild	12 (37.5%)	10 (47.6%)			
Moderate	1 (3.1%)	10 (47.6%)			
Severe	0 (0.0%)	1 (4.8%)			
Delayed phase			< 0.001 ^a^	0.000	0.913
Minimal	17 (53.1%)	0 (0.0%)			
Mild	14 (43.8%)	10 (47.6%)			
Moderate	1 (3.1%)	10 (47.6%)			
Severe	0 (0.0%)	1 (4.8%)			

Except where otherwise indicated, data are the number (%) of patients. *ADC* apparent diffusion coefficient, *DWI* diffusion-weighted imaging, *LASSO* least absolute shrinkage and selection operator, *OTFG* ovarian thecoma-fibroma group, *OGCT* ovarian granulosa cell tumor, *OR* odds ratio, *SD* standard deviation, *T1WI* T1-weighted imaging, *T2WI* T2-weighted imaging

^a^Data were tested using the Mann–Whitney U test

^b^The intra-class correlation (ICC) was calculated instead because of continuous variables

All variables were included in the LASSO regression analysis. To establish a discriminating model using representative MBFs, we selected 3 features with the highest absolute value of the LASSO regression coefficient: the ADC value, peripheral cystic area, and contrast enhancement in the venous phase (VCE). The AUC of the discriminating model using these representative MBFs was 0.938, with an accuracy of 0.905, sensitivity of 0.857, and specificity of 0.937 (Table 6).

We used Kappa values to investigate interobserver variability and found that the observers were in agreement on most of the conventional MR features. Kappa values ranged between 0.76 and 0.96, as shown in Tables 3 and 4. The collinearity status between each feature is shown in Supplementary Fig. 1.

### TA

**Fig. 4:**
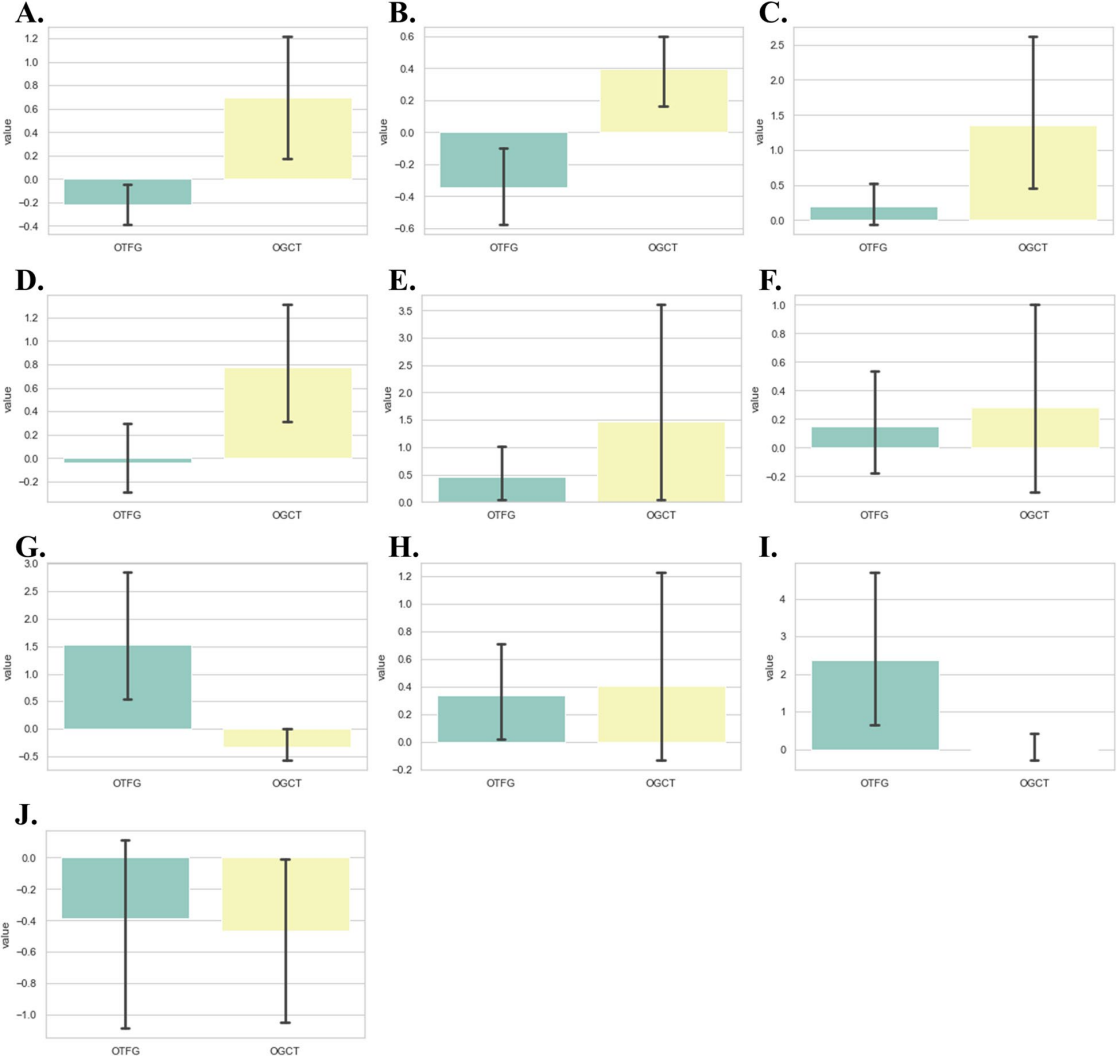
Bar charts showing the distribution of 10 selected texture features (TFs), with the ovarian thecoma-fibroma group (OTFG) denoted by a green bar and the ovarian granulosa cell tumor group (OGCT) by a yellow bar. The name codes of the selected TFs are as follows: difference variance (**a**), 10th percentile (**b**), large area high gray-level emphasis (**c**), strength (**d**), zone variance (**e**), joint energy (**f**), busyness (**g**), total energy (**h**), short run high gray-level emphasis (**i**), and maximal correlation coefficient (**j**)

The mean sizes of the ROIs placed by two radiologists (T.K. and F.Y.) were 118.31 ± 86.78 and 125.19 ± 92.43 cm^2^, respectively. Among 93 TFs, the mean ICC value was 0.864 ± 0.124 in the inter-observer reproducibility test. The number of features with good reproducibility (ICC ≥ 0.75 and lower 95%CI ≥ 0.6) was 80. All variables were included in the LASSO regression analysis. TFs with LASSO regression coefficients other than 0 were summarized in Table 5 and their distribution was shown in Fig. 4. To establish a discriminating model using representative TFs, we selected 3 features with the highest absolute values of LASSO regression coefficients: the histogram-based 10th percentile, GLCM-based difference variance, and maximal correlation coefficient. The AUC of the discriminating model using these representative TFs was 0.817, with an accuracy of 0.791, sensitivity of 0.618, and specificity of 0.904 (Table 6). The collinearity status between each feature is shown in Supplementary Fig. 1.

**Table 5:** Representative texture features selected using the LASSO algorithm

Feature name code	Value*, mean ± SD		Univariate analysis (*p*)	LASSO estimate	ICC
	OTFG	OGCT			
Diff.variance	-0.20 ± 0.48	0.64 ± 1.17	0.012	0.163	0.757
10th percentile	-0.34 ± 0.65	0.38 ± 0.52	< 0.001	0.104	0.988
LAHGLE	0.18 ± 0.80	1.28 ± 2.40	0.015	0.056	0.792
Strength	-0.01 ± 0.87	0.72 ± 1.14	0.007	0.040	0.578
Zone variance	0.43 ± 1.36	1.39 ± 4.38	0.993	0.027	0.944
Joint energy	0.13 ± 1.01	0.34 ± 1.57	0.834	0.002	0.950
Busyness	1.46 ± 3.21	-0.29 ± 0.67	< 0.001	-0.007	0.806
Total energy	0.30 ± 1.04	0.35 ± 1.61	0.617	-0.018	0.952
SDLGLE	2.31 ± 6.20	0.03 ± 0.84	0.003	-0.025	0.991
MCC	-0.43 ± 1.74	-0.70 ± 1.58	0.248	-0.088	0.779

All 93 texture features (TFs) were included in the LASSO regression analysis. Features with a coefficient other than 0 are shown in this table. Regarding all variables, numeric values were standardized by RobustScaler before the statistical analysis (* standardized values are shown). *ICC* intraclass correlation coefficient, *LASSO* least absolute shrinkage and selection operator, *SD* standard deviation, *OTFG* ovarian thecoma-fibroma group, *OGCT* ovarian granulosa cell tumor. Feature name codes are as follows: *Diff.variance* difference variance, *LAHGLE* large area high gray-level emphasis, *MCC* maximal correlation coefficient, *SDLGLE* small dependence low gray-level emphasis

### Combination of conventional MRI-based characteristics and TA

We evaluated the performance of the diagnostic model using the best parameters of MRI-based characteristics and TA. All MBFs and TFs were included in the LASSO regression analysis. Features with LASSO regression coefficients other than 0 are summarized in Supplementary Table 1. We selected the best 3 features with the highest absolute value of the LASSO regression coefficient: ADC, VCE, and difference variance. The AUC of the discriminating model using these representative features was 0.941, with an accuracy of 0.901, sensitivity of 0.893, and specificity of 0.906 (Table 6).

**Table 6:** Performance of each individual feature in OTFG and OGCT classifications

	Accuracy	Sensitivity	Specificity	F-measure	AUC
MBF					
ADC	0.792	0.644	0.888	0.710	0.823
PC	0.679	0.952	0.500	0.702	0.640
VCE	0.792	0.524	0.969	0.667	0.843
Representative MBFs					
ADC + PC + VCE	0.905	0.857	0.937	0.878	0.938
TF					
10th pct	0.759	0.632	0.843	0.675	0.809
Diff.var	0.774	0.524	0.938	0.647	0.691
MCC	0.588	0.015	0.964	0.027	0.491
Representative TFs					
10th pct + Diff.var + MCC	0.791	0.618	0.904	0.700	0.817
MBFs with TFs					
VCE + Diff.var + ADC	0.901	0.893	0.906	0.877	0.941

All data are mean values. *AUC* area under the curve, *OTFG* ovarian thecoma-fibroma group, *OGCT* ovarian granulosa cell tumor, *MBF* MRI-based feature, *TF* texture feature. Feature name codes are as follows: *10th pct* 10th percentile, *ADC* apparent diffusion coefficient, *Diff.var* difference variance, *MCC* maximal correlation coefficient, *PC* peripheral cystic area, *VCE* venous phase contrast enhancement

### Comparison of representative classification models

**Fig. 5:**
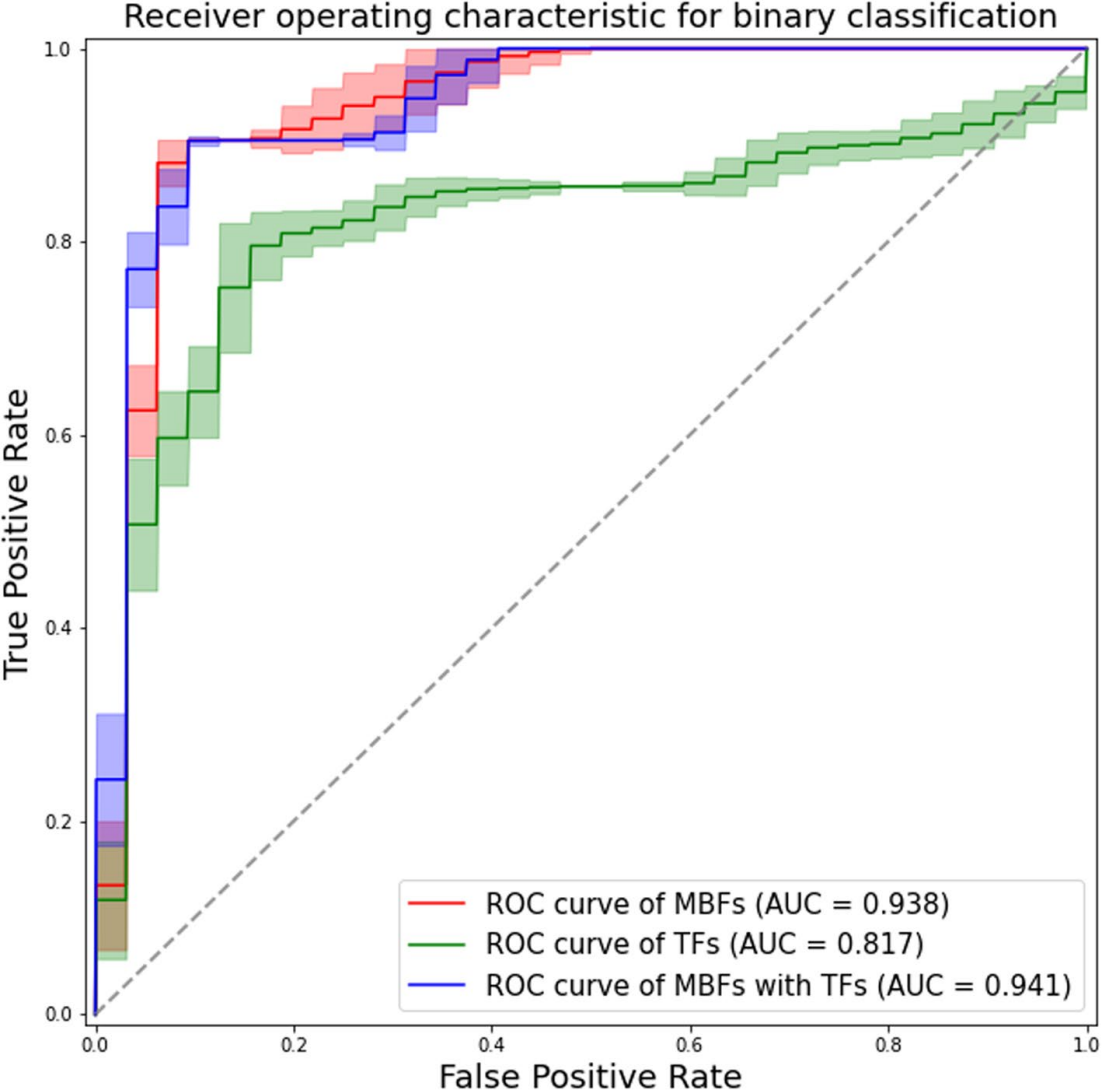
ROC curves and AUC values of classification attempts for differentiating between ovarian thecoma-fibroma groups (OTFGs) and ovarian granulosa cell tumors (OGCTs) using representative MRI-based features (MBFs) denoted by a red line, representative texture features (TFs) denoted by a green line, and the combination of MBFs and TFs denoted by a blue line

The performance of representative classification models (MRI-based, TA-based, and combination models) was compared using bootstrap likelihood ratio tests. The AUCs for these models in each of 100 bootstrap samples are shown in Supplementary Table 2. The diagnostic performance of the MRI-based and combination models was similar (*p* = 0.38), but significantly better than that of the TA-based model (*p* < 0.05). All results are summarized in Table 6, with representative ROC curves in Fig. 5.

## Discussion

In the present study, we assessed the ability of a conventional MRI-based analysis and TA to distinguish between OGCTs and OTFGs. We found that several MBFs were important for differentiating between the two disease groups. The peripheral cystic area, ADC values, and VCE were the most valuable differentiators. We also showed that TA may be helpful for distinguishing between OGCTs and OTFGs, but were unable to identify its advantages over MBFs in the present study.

Previous studies reported that conventional MRI findings may facilitate the differentiation of OGCTs from OTFGs [8–12]. An assessment of the cystic component is important for the diagnosis of OTFGs and OGCTs. In the present study, cystic areas were more frequently detected in OGCTs than in OTFGs. The maximum diameter of cysts was similar between the two groups in the present study; however, a significant difference was reported in a previous study [10]. We showed that the presence of peripheral cystic areas may be an important indicator in the differential diagnosis of OTFGs and OGCTs. In OTFGs, cystic changes are mainly caused by degeneration, and occur in the peripheral zone in approximately 50% of cases [11, 21]. Regarding OGCTs, the present results demonstrated that cystic areas were always present peripherally and more frequently than in OTFGs, and contralateral or distal to the adnexal vascular inflow site in most cases. The frequency of capsules was previously reported to be significantly higher in OGCTs than in OTFGs [8, 10, 11, 24], and this was confirmed in the present study. Morphology was also examined in a previous study, with OTFGs showing multiple nodular lesions and OGCTs mostly exhibiting oval lesions [10]. However, in the present study, no significant differences were observed between the two groups. Hemorrhage is a distinctive feature in OGCTs [25]. In the present study, a hemorrhagic component was observed in 61.9% of cases, which was consistent with previous findings [10, 25].

On T2WI in the present study, more than 50% of OGCTs showed hyperintense signals, while OTFGs had various signal intensities. OTFGs in conventional MRI findings often had a low signal intensity on T2WI, representing the fibrous component of the tumor [10]. However, the extent of the low signal may be affected by edema, degenerative changes, or the amount of theca cells and fibrous content, resulting in various degrees of signal intensity [11]. On DWI in the present study, OGCTs and OTFGs both exhibited a high or mixed signal intensity. The ADC value of the solid component was significantly higher in OTFGs than in OGCTs. DWI reflects the motion of water molecules, and water diffusivity may be quantitatively calculated using the ADC value. Increased cellularity in lesions generally causes the ADC value to decrease, and this has potential for differentiating between benign and malignant lesions[26]. Regarding OGCTs and OTFGs, the ADC value may be affected by the degree of cellularity (the number of granulosa cells or fibroblasts), minor degeneration, or edematous changes. Previous studies reported that a high density of granulosa cells in OGCTs may be related to reduced values, while the likelihood of degeneration in OTFGs may result in an increase in the ADC value; however, there is an overlap between the two diseases [8, 10, 27].

OGCTs and OTFGs have been suggested to have different blood supplies, with the former being more hypervascular than the latter [8, 10]. In the present study, OTFGs always exhibited minimal to mild enhancement, while OGCTs mostly showed mild to moderate enhancement. Regarding contrast phases, according to one study, the distinction between thecomas and OGCTs was more apparent in the venous and delayed periods than in the arterial period of multiphasic contrast enhancement [10]. These findings were confirmed in the present study, with VCE being identified as a more significant independent differentiator of the two disease groups than that in the other phases. This result may be attributed to the variability of enhancement degrees in lesions as well as in the myometrium (used as a reference), which may be emphasized in the early contrast phase.

The present results implied that the extent and pattern of signal intensities differ between the two disease groups. Although it is of high clinical value for these differences to be made explicit, difficulties are associated with assessing them in a quantitative manner. In comparisons with visual assessments of texture, computational TA techniques are reportedly more objective and sensitive to changes that are imperceptible to the human visual system [13, 14]. In the present study, the following TFs on T2WI were particularly important for differentiating between OGCTs and OTFGs: the 10th percentile (a histogram-based value below which 10% of all data in a set fall) and the difference variance (a GLCM-based feature that measures the deviation from the mean value) [28–30]. OTFGs showed a lower 10th percentile, indicating a predominant area with a low signal intensity (fibrous component). In contrast, OGCTs displayed a higher signal intensity (granulosa cell component) and higher difference variance (implying heterogeneity caused by hemorrhage or fibrosis).

The present results revealed that the diagnostic performance of the MRI-based and combination models was similar, and no additional benefit was obtained in TA. These results were contradictory to recent findings reported by Li et al. showing that TA-based and combination models were superior to the MBF-based model [22]. However, there were important differences between that study and the present study; (1) The number of parameters they initially applied in TA was 1,316, which was too big for the small sample size of their study (46 cases). It may have led to over-fitting, even if they used multi-step feature selection including the LASSO algorithm. (2) The present study was conducted at two institutions, whereas theirs was a single-center study. Although we performed image preprocessing and data standardization, variations between the two centers may have resulted in inferior results for TA. (3) The present study considered the solid portion of the lesion in only one slice of axial T2WI for TA instead of the whole area throughout all slices of the tumor in their study. Therefore, the extracted data may have differed, and their data may have contained more texture information than ours.

The present study had a number of limitations that need to be addressed. The sample size was small because the rarity of these disorders prevented us from collecting a large number of patients. In addition, this study was retrospective, which inevitably increases the risk of bias. Future prospective studies with a large sample size and independent training and test cohorts will further underline the diagnostic value of the present results. Moreover, the present study lacked other ovarian tumors (particularly other sex-cord tumors, such as sclerosing stromal tumors). Another limitation is that our TA study only considered the solid region of the tumor on each selected image, while the cystic region was avoided as much as possible. By omitting these regions, we may have lost some important TA information. Similarly, we only used T2WI axial images for TA in the present study. We did not include additional TA on T1WI or contrast-enhanced images because these sequences were sometimes taken in only the coronal, sagittal, or oblique plane, and axial images were not available in all patients. Furthermore, as discussed above, we delineated the ROI in a single slice because it contained representative information on tissue texture and may be a substitute for all slice data. This methodology has been applied in many studies on TA. Furthermore, it is time-consuming to delineate all slices. However, in a recent study, good findings were obtained for TA-based models using all slice data [22]; therefore, further studies are needed. The further development of our models will be achieved by addressing these issues in the future.

## Conclusions

The conventional MRI-based analysis may be used to differentiate OTFGs from OGCTs. TA did not appear to have any additional benefits. Further studies are needed to verify their reproducibility and feasibility for the non-invasive preoperative diagnosis of these two diseases.

## Supplementary Information

**Additional file 1.**

## Data Availability

The authors declare that all data supporting the results of the present study are available within the article.

## Abbreviations

ADC: Apparent diffusion coefficient
AUC: Area under the curve
DWI: Diffusion-weighted imaging
GLCM: Gray-level co-occurrence matrix
ICC: Intraclass correlation coefficient
LASSO: Least absolute shrinkage and selection operator
MBF: MRI-based feature
MRI: Magnetic resonance imaging
OGCT: Ovarian granulosa cell tumor
OTFG: Ovarian thecoma-fibroma group
ROI: Region of interest
TA: Texture analysis
TF: Texture feature
