# Supplementary material for: Diagnostic utility of a conventional MRI-based analysis and texture analysis for discriminating between ovarian thecoma-fibroma groups and ovarian granulosa cell tumors
Source: J Ovarian Res. 2022 May 25;15:65. doi: 10.1186/s13048-022-00989-z (PMC9131674; doi:10.1186/s13048-022-00989-z)
Supplement: Supplementary file 1 — Additional file 1. [file 13048_2022_989_MOESM1_ESM.docx]

Supplementary Table 1. Representative MRI-based features with texture features selected by the LASSO algorithm

Feature name code LASSO estimate

Venous phase contrast enhancement (MBF) 0.255

Difference variance (TF) 0.113

Diffusion-weighted image (MBF) 0.049

Delayed phase contrast enhancement (MBF) 0.041

Minimum (TF) 0.038

Strength (TF) 0.038

Arterial phase contrast enhancement (MBF) 0.030

Large area high gray-level emphasis (TF) 0.020

Peripheral cystic area (MBF) 0.018

Joint energy (TF) 0.015

10th percentile (TF) 0.009

Zone variance (TF) 0.008

Busyness (TF) -0.003

Total energy (TF) -0.013

Small dependence low gray-level emphasis (TF) -0.026

Apparent diffusion coefficient (MBF) -0.065

Note— All MRI-based features (MBFs) and texture features (TFs) were included in the LASSO regression analysis. Variables with a coefficient other than 0 were selected as representative parameters and are shown in this table. Regarding all variables, numeric values were standardized by RobustScaler before the statistical analysis. LASSO, least absolute shrinkage and selection operator.

Supplementary Table 2. AUC values of representative classification models in each of 100 bootstrap samples

Time MBFs TFs MBFs with TFs

1 0.930 0.823 0.939

2 0.933 0.814 0.945

3 0.938 0.810 0.940

4 0.933 0.808 0.939

5 0.949 0.820 0.943

6 0.930 0.818 0.936

7 0.936 0.815 0.943

8 0.930 0.824 0.936

9 0.935 0.798 0.940

10 0.936 0.821 0.936

11 0.942 0.821 0.943

12 0.935 0.817 0.942

13 0.942 0.804 0.942

14 0.946 0.824 0.940

15 0.932 0.810 0.942

16 0.945 0.821 0.940

17 0.942 0.823 0.943

18 0.923 0.830 0.940

19 0.938 0.814 0.943

20 0.952 0.815 0.948

21 0.943 0.807 0.938

22 0.946 0.823 0.945

23 0.942 0.818 0.938

24 0.939 0.792 0.939

25 0.945 0.817 0.945

26 0.939 0.824 0.940

27 0.940 0.823 0.940

28 0.929 0.808 0.939

29 0.936 0.817 0.939

30 0.946 0.827 0.945

31 0.936 0.823 0.935

32 0.933 0.814 0.943

33 0.938 0.814 0.939

34 0.938 0.814 0.936

35 0.945 0.823 0.946

36 0.930 0.820 0.938

37 0.946 0.830 0.945

38 0.935 0.814 0.948

39 0.945 0.808 0.935

40 0.940 0.823 0.940

41 0.948 0.833 0.940

42 0.936 0.829 0.935

43 0.929 0.805 0.942

44 0.936 0.821 0.943

45 0.932 0.832 0.939

46 0.938 0.815 0.932

47 0.942 0.807 0.936

48 0.929 0.798 0.942

49 0.938 0.811 0.943

50 0.942 0.813 0.943

51 0.940 0.814 0.945

52 0.933 0.827 0.940

53 0.926 0.823 0.940

54 0.929 0.813 0.940

55 0.943 0.824 0.936

56 0.949 0.813 0.936

57 0.948 0.823 0.945

58 0.936 0.821 0.942

59 0.938 0.818 0.940

60 0.943 0.824 0.936

61 0.936 0.820 0.942

62 0.930 0.810 0.936

63 0.933 0.832 0.939

64 0.938 0.818 0.933

65 0.942 0.820 0.943

66 0.936 0.811 0.939

67 0.932 0.804 0.936

68 0.936 0.829 0.945

69 0.938 0.818 0.942

70 0.942 0.827 0.945

71 0.943 0.818 0.935

72 0.923 0.829 0.939

73 0.935 0.814 0.940

74 0.943 0.815 0.945

75 0.935 0.833 0.945

76 0.927 0.826 0.943

77 0.939 0.805 0.935

78 0.943 0.817 0.943

79 0.939 0.824 0.946

80 0.942 0.802 0.943

81 0.929 0.820 0.942

82 0.935 0.814 0.948

83 0.929 0.811 0.940

84 0.926 0.829 0.940

85 0.945 0.802 0.942

86 0.929 0.817 0.933

87 0.938 0.821 0.940

88 0.940 0.804 0.943

89 0.946 0.829 0.943

90 0.939 0.829 0.939

91 0.946 0.826 0.939

92 0.938 0.807 0.938

93 0.938 0.807 0.938

94 0.951 0.813 0.940

95 0.927 0.824 0.940

96 0.943 0.820 0.942

97 0.939 0.826 0.943

98 0.936 0.814 0.939

99 0.935 0.815 0.942

100 0.936 0.811 0.943

Note— AUC, area under the curve; MBF, MRI-based features; TF, texture feature.


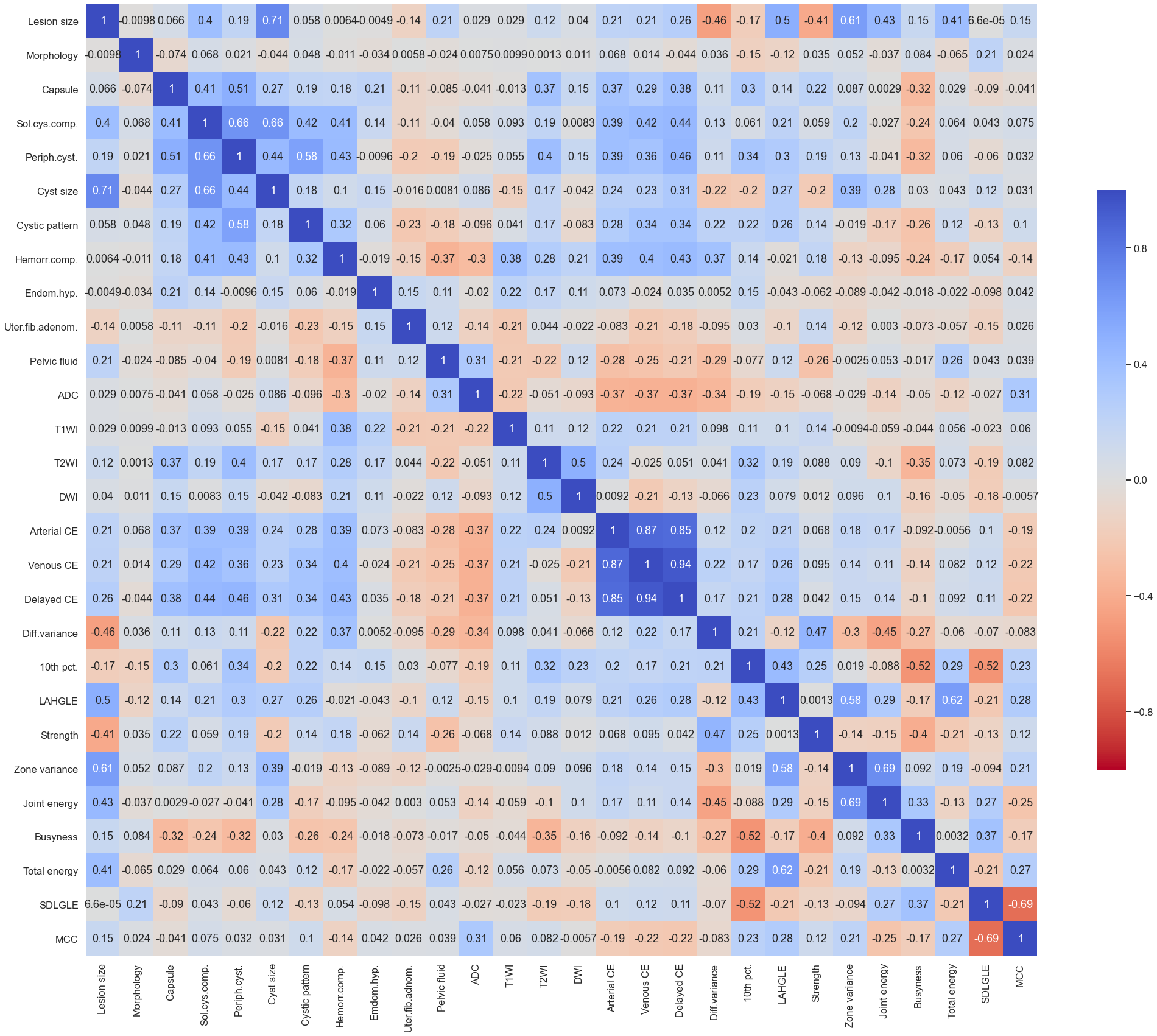


Supplementary Figure 1. Correlation matrix showing the relationship between each MRI-based feature (MBF)/texture feature (TF).
